# TMEM16A, a Homoharringtonine Receptor, as a Potential Endogenic Target for Lung Cancer Treatment

**DOI:** 10.3390/ijms222010930

**Published:** 2021-10-10

**Authors:** Shuai Guo, Xue Bai, Sai Shi, Yawen Deng, Xianjiang Kang, Hailong An

**Affiliations:** 1 School of Life Science, Hebei University, Baoding 071002, China; 2 Key Laboratory of Molecular Biophysics, Institute of Biophysics, School of Sciences, Hebei University of Technology, Tianjin 300401, China; shisaicn@163.com (S.S.); hailong_an@hebut.edu.cn (H.A.); 3 School of Pharmacy, Hebei University, Baoding 071002, China; d17330243114@163.com

**Keywords:** TMEM16A, homoharringtonine, drug target, inhibitor, lung adenocarcinoma

## Abstract

Lung cancer has the highest rate of incidence and mortality among all cancers. Most chemotherapeutic drugs used to treat lung cancer cause serious side effects and are susceptible to drug resistance. Therefore, exploring novel therapeutic targets for lung cancer is important. In this study, we evaluated the potential of TMEM16A as a drug target for lung cancer. Homoharringtonine (HHT) was identified as a novel natural product inhibitor of TMEM16A. Patch-clamp experiments showed that HHT inhibited TMEM16A activity in a concentration-dependent manner. HHT significantly inhibited the proliferation and migration of lung cancer cells with high TMEM16A expression but did not affect the growth of normal lung cells in the absence of TMEM16A expression. In vivo experiments showed that HHT inhibited the growth of lung tumors in mice and did not reduce their body weight. Finally, the molecular mechanism through which HHT inhibits lung cancer was explored by western blotting. The findings showed that HHT has the potential to regulate TMEM16A activity both in vitro and in vivo and could be a new lead compound for the development of anti-lung-cancer drugs.

## 1. Introduction

Lung cancer has the highest incidence and mortality rates worldwide among all cancers [1]. According to estimates, the incidence of lung cancer will linearly increase over the next 20 years [2,3]. Therefore, prevention and treatment of lung cancer is important. Currently, surgery, chemotherapy, and radiotherapy are the main treatment options for lung cancer; however, all of these options have disadvantages: surgery is risky and restrictive [4,5], chemotherapy is not completely effective and can lead to drug resistance [6], and radiotherapy is associated with serious side effects [7]. The five-year survival rates of patients with stage IA, IB, IIA, IIB, IIIA, IIIB, and IV lung cancers are 73%, 58%, 46%, 36%, 24%, 9%, and 2%, respectively [8]. Therefore, identifying novel biomarkers for early diagnosis and targeted therapy is important for the prevention and treatment of lung cancer [9].

TMEM16A is a calcium-activated chloride channel (CaCC) with important physiological functions [10,11]. TMEM16A is widely expressed in epithelial and smooth muscle tissues as well as in various glands of the human body [12]. Studies have shown that TMEM16A (also known as ANO1, DOG1, TAOS2, or ORAOV2) is associated with several cancer types [13,14]. The TMEM16A protein is highly expressed in oral, esophageal, lung, liver, and prostate cancers; its overexpression is closely related to the proliferation and migration of cancer cells [15,16]. In addition, clinical data indicate that TMEM16A is also significantly associated with poor prognosis in some cancers [17]. Several recent studies have shown that inhibiting the overexpression of TMEM16A in lung cancer impedes tumor evolution [18]. Therefore, TMEM16A has emerged as a potential drug target for lung cancer treatment [19].

Homoharringtonine (HHT) is an alkaloid isolated from plants (conifers) of the *Cephalotaxaceae* family [20]. It is clinically used to treat chronic myelogenous leukemia (CML), acute myeloid leukemia (AML), and malignant lymphoma [21,22]. However, the molecular mechanisms underlying the anti-cancer effects of HHT are not clear. Studies have shown that HHT inhibits cancer cell proliferation by inhibiting protein and DNA syntheses [23]. The lethality of HHT against G1 and G2 phase cells is strong, but the effect on S phase cells is weak. In CML, HHT prevents the elongation step of protein synthesis by interacting with the A-site of the ribosome and disrupting the positioning of aminoacyl-tRNAs [24]. In breast cancer, HHT suppresses cell growth and promotes apoptosis by regulating the miR-18a-3p-AKT-mTOR signaling pathway [25]. In FLT3-ITD AML, HHT induces cancer cell apoptosis through inhibiting the FLT3-AKT-c-Myc pathway [26]. Although there have been several studies on HHT anti-cancer properties, the HHT receptors on lung cancer cells and the downstream signal transduction mechanisms related to HHT interaction with cells are still unclear. Therefore, the mechanisms associated with cancer suppression by HHT should be studied further.

In this study, we evaluated the inhibitory effects of HHT on lung cancer cells via targeting TMEM16A and explored its anti-cancer mechanisms. This study may provide preliminary guidance on developing chemotherapeutic drugs with HHT as the lead compound for lung cancer treatment.

## 2. Materials and Methods

### 2.1. TCGA Data Mining

RNA-seq and clinical data from patients with lung adenocarcinoma were obtained from the TCGA database. TMEM16A expression data were normalized; the clinical data for each sample were acquired. A cutoff value of 13.3000 was selected for normalized TMEM16A expression according to the ROC analysis for overall survival status. A total of 50 and 30 tissue samples were randomly selected for clinical stage and lymph node metastasis analyses, respectively.

### 2.2. Cell Culture

LA795 and NCI-H1299 cells were cultured in RPMI 1640 (Solarbio, Beijing, China). A549 and 2BS cells were maintained in F12K (2850 Grand Island Blvd, Grand Island, NY, USA) and DMEM (Solarbio), respectively. The medium was supplemented with fetal bovine serum (10%) (Sijiqing, Hangzhou, China), 100 UI/mL penicillin (Solarbio), and 100 μg/mL streptomycin (Solarbio). All the cells were cultured under standard conditions of 5% CO_2_ and 95% humidity at 37 °C and passaged every 2 days. TMEM16A or shRNA plasmid were transfected into cells as described previously with X-tremeGENE HP (Roche, Switzerland) [27]. The following shRNA targeting the TMEM16A gene was used: CCTGCTAAACAACATCATT (2399–2418 nt).

### 2.3. Western Blot Analysis

The cells were collected and lysed using pre-cold RIPA buffer. The isolated proteins were separated on 10% sodium dodecyl sulfate-polyacrylamide gel electrophoresis and electroblotted onto a nitrocellulose membrane in 25 mM Tris base and 190 mM glycine at 100 V for 2 h. The blots were incubated for 8 h at 4 °C in 1:1000 dilution of the corresponding primary monoclonal antibodies against TMEM16A (ab53212, Abcam, Cambridge, UK), MEK1/2 (ab178876, Abcam), p-MEK1/2 (11205, Signalway, TX, USA), ERK1/2 (K200062M, Solarbio), p-ERK1/2 (12548, Signalway), cyclin D1 (60186-1-Ig, Proteintech, Chicago, IL, USA), cleaved-caspase 3 (AF7022, Affinity Biosciences, Changzhou, China), cleaved-caspase 9 (AF5240, Affinity Biosciences), β-catenin (ab223075, Abcam), *N*-cadherin (A01577-3, Boster, Beijing, China), E-cadherin (BM4166, Boster), and vimentin (10366-1-AP, Proteintech). This step was followed by incubation with horseradish peroxidase-conjugated goat anti-rabbit (IgG) secondary antibody (ab150077, Abcam) for 1 h at 37 °C. Blots were detected using an enhanced chemiluminescence detection kit (BIO-RAD, Hercules, CA, USA).

### 2.4. Electrophysiology

During the whole-cell patch-clamp experiments, recordings were obtained using an EPC10 amplifier controlled by Patchmaster software with a Digi LIH1600 interface (HEKA, Lambrecht, Germany). Data were low-pass filtered at 2.9 kHz and sampled at 10 kHz. The stimulation protocol included voltage steps with a duration of 1200 ms from a holding potential of 0 mV. The membrane voltage (Vm) was clamped in steps of 20 mV from −80 mV to +80 mV, followed by −80 mV. The pipette resistance was 3–5 MΩ when it was immersed in a bath solution that was drawn using a P-97 puller (Sutter Instruments, Novato, CA, USA).

The pipette solution contained 130 mM CsCl, 10 mM EGTA, 1 mM Mg-ATP, 1 mM MgCl_2_·6H_2_O, and 10 mM HEPES; the solution was adjusted to pH 7.4 using CsOH. The bath solution contained 150 mM NaCl, 1 mM MgCl_2_·6H_2_O, 10 mM HEPES, 10 mM glucose, and 10 mM mannitol; the solution was adjusted to pH 7.4 using NaOH. The osmolality of the solution was determined using an OM815 osmometer (Löser Messtechnik, Berlin, Germany), with 290–300 mOsm/L for the pipette solution and 300–310 mOsm/L for the bath solution.

### 2.5. Molecular Docking

The calcium-bound mTMEM16A chloride channel (PDB ID: 5oyb) was used to construct the tertiary structure of the mTMEM16A monomer [28]. The missing structure of 5oyb was complemented by SWISS-MODEL [29]. The AutoDock 4.2 program was used to perform the binding sites of HHT to mTMEM16A through the implemented empirical free energy function and the Lamarckian Genetic Algorithm (LGA). A two-step docking strategy was used for molecular docking. Global random docking was performed 100 times without any restrictions. Next, a 35 × 35 × 35 Å domain was selected for local docking, which was centered on the area where HHT was most distributed in the global random docking. A root mean square (RMS) tolerance of 2.0 Å was adopted to perform cluster analysis on the docked results; ChemBioDraw Ultra 12.0 was used to map the initial coordinates of the ligand. Additionally, VMD1.9 and Pymol 1.1 were used for visualization and analysis of the complex, respectively.

### 2.6. Site-Directed Mutagenesis

Site-directed mutagenesis primer was designed using the Agilent primer design website (https://www.agilent.com/store/primerDesignProgram.jsp, accessed on 18 June 2018). The primer for K769A (5′-catcctcagaggtgttggggcgctggctgtcatcattaat-3′) was synthesized by Sangon Biotech (Shanghai, China). Site-directed mutagenesis was conducted using a Fast Mutagenesis System Kit (FM111-02, Transgen, Beijing, China) at a 50 μL reaction volume. The mutated plasmid was sequenced by Sangon Biotech (Shanghai, China).

### 2.7. CCK-8 Assay

LA795 and 2BS cells were seeded in 96-well plates at a density of 4000–7000 cells/well to detect cell proliferation. The cells were cultured for 24 h and then treated with the indicated concentrations of HHT for 24 h, followed by CCK-8 solution (Solarbio, Beijing, China) for 2 h. Absorbance was measured at 450 nm using a microplate reader (SpectraMAX i3, Molecular Devices, Sunnyvale, CA, USA).

### 2.8. Wound Healing Assay

LA795 and 2BS cells were cultured to 90% confluence in a 6-well plate and scraped with a sterile 10 μL micropipette tip. The normal medium was replaced with fresh medium containing 1% FBS and different concentrations of HHT. Images were acquired at 0, 24, 48, and 72 h using an inverted microscope (100× magnification; Nikon, Tokyo, Japan). The wound healing area was calculated using ImageJ software (National Institutes of Health, Bethesda, MD, USA). The percentage of relative scratch area was determined based on the ratio of the average unoccupied area in the drug-treated cells to that in the control groups.

### 2.9. Annexin V Assay

Cell apoptosis was detected using the Annexin V-FITC Apoptosis Detection Kit (CA1020, Solarbio). LA795 cells were seeded into 6-well culture plates for 24 h. Next, the cells were incubated with HTT (30 μM) for 24 h. The cells were trypsinized and suspended in 500 µL of binding buffer containing 5 µL Annexin V-FITC and 5 µL propidium iodide (PI). Finally, the cells were analyzed using a CytoFLEX flow cytometer (Beckman Coulter, Brea, CA, USA).

### 2.10. Tumor Xenografts in Mice

All animal experiments were conducted in accordance with the approved guidelines of the Ethical Review Committee of Experimental Animal Welfare, Hebei University (license No. SCXK (Ji) 2017-002). Accordingly, 5 × 10^6^ LA795 cells were inoculated into the right forelimb of 6- to 8-week-old BALB/c mice (SPF Biotechnology Co., Ltd., Beijing, China). The length and width of the tumor were measured using a Vernier caliper every 3 days. The standard formula (length × width^2^/2) was used to calculate the tumor volume. The mice were divided into four groups (*n* = 6 per group): (1) control group (injected with normal saline), (2) cisplatin group (10 mg/kg body weight [BW]/3d), (3) HHT group (15 mg/kg/kg body weight (BW)/3d), and (4) HHT group (25 mg/kg/kg body weight (BW)/3d). All mice were subcutaneously injected with drugs every 3 days and were sacrificed after 10 injections.

### 2.11. Data Analysis

Statistical data were analyzed using Origin 8.0, and the graphics were created using GraphPad Prism 8. All data are presented as the mean ± SE. Statistical significance between two groups was determined using ANOVA and an independent *t*-test. Asterisks indicate significant differences (* *p* < 0.05, ** *p* < 0.01). The capacitive transients of some traces in the figures were trimmed for clarity.

## 3. Results

### 3.1. TMEM16A Is Highly Expressed in Lung Adenocarcinoma Cells

**Figure 1:**
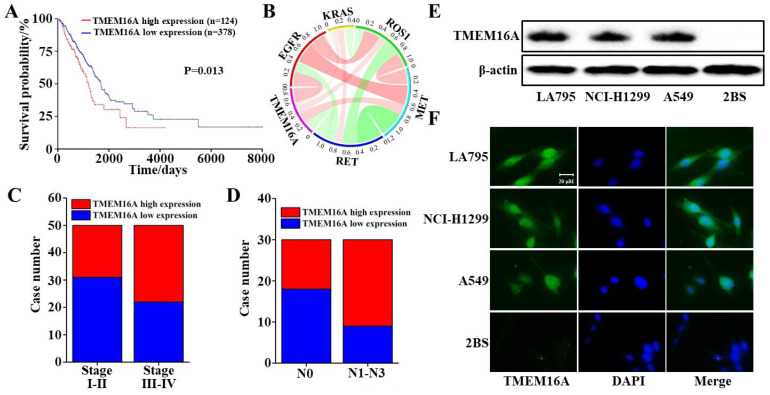
TMEM16A was highly expressed in malignant lung adenocarcinoma. (**A**) Survival time curve of lung adenocarcinoma patients with high or low expression of TMEM16A. (**B**) Correlation analysis between TMEM16A overexpression and EGFR, KRAS, ROS1, MET, and RET. (Red: positive correlation. Green: negative correlation. The absolute value of the width represents the correlation coefficient.) (**C**) The proportion of patients with high or low TMEM16A expression in lung adenocarcinoma stages Ⅰ–Ⅱ and stages Ⅲ–Ⅳ. (**D**) The proportion of patients with high or low expression of TMEM16A in N0 and N1–N3 stages of lung adenocarcinoma lymph node metastasis. (**E**,**F**) Expression of TMEM16A in LA795, NCI-H1299, A549, and 2BS cells tested by western blot and immunofluorescence.

The relationship between TMEM16A expression and the survival rate of 502 samples from patients with lung adenocarcinoma from the TCGA database was analyzed. The results showed that the survival time of patients with lung adenocarcinoma with low TMEM16A expression was significantly longer than that of patients with high TMEM16A expression (Figure 1A). In addition, correlation analysis of 585 sample data showed that TMEM16A overexpression is positively correlated with EGFR, KRAS, ROS1, and MET, and negatively correlated with RET (Figure 1B). The relationship between TMEM16A expression and the clinicopathological characteristics of patients with lung adenocarcinoma in the TCGA database was also analyzed. The results showed that the expression of TMEM16A was significantly related to the clinical stage in patients with lung adenocarcinoma, in which the expression of TMEM16A was higher at stages III and IV than at stages I and II (Figure 1C). In addition, the expression of TMEM16A in patients with lymph node metastasis at stages N1–N3 was higher than those at stage N0 (Figure 1D). TMEM16A expression was detected in the lung cancer cell lines LA795, NCI-H1299, and A549 as well as in the human fetal lung diploid fibroblast cell line 2BS. Western blotting and immunofluorescence analyses showed that TMEM16A was highly expressed in LA795, NCI-H1299, and A549 cells, but not in 2BS cells (Figure 1E,F). In summary, TMEM16A was highly expressed in lung adenocarcinoma cells and was related to patient survival time, tumor stage, and tumor metastasis.

### 3.2. TMEM16A Currents Are Inhibited by HHT in a Concentration-Dependent Manner

**Figure 2:**
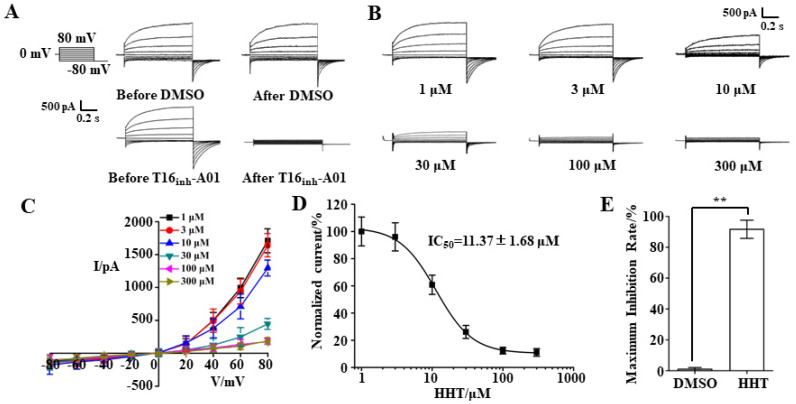
HHT inhibited TMEM16A whole-cell current in LA795 cells. (**A**) Typical TMEM16A whole-cell currents of DMSO and 16A_inh_-A01 perfusion in LA795 cells (*n* = 5). (**B**) Typical TMEM16A whole-cell currents inhibited by different concentrations of HHT (*n* = 5). (**C**) I–V curve of the TMEM16A currents inhibited with different concentrations of HHT (*n* = 5). (**D**) Dose-response curve for HHT inhibition of TMEM16A currents in LA795 cells (*n* = 5). (**E**) Statistical results of the maximum inhibition rate of HHT on LA795 whole-cell currents (*n* = 5, ** *p* < 0.01).

The TMEM16A-specific inhibitor, T16A_inh_-A01, was used to verify TMEM16A whole-cell currents in LA795 cells. The currents in these cells activated by 600 nM Ca^2+^ were completely inhibited by 10 μM T16A_inh_-A01 (Figure 2A). A whole-cell patch-clamp experiment was performed to detect the inhibitory effect of HHT on TMEM16A. The results of the HHT perfusion experiment with different concentrations showed that 1 μM HHT hardly inhibited TMEM16A currents; the inhibitory efficiencies of 3 μM, 10 μM, and 30 μM HHT on TMEM16A currents were 4.0%, 24.2%, and 73.9%, respectively. More than 100 μM HHT almost completely inhibited TMEM16A currents (Figure 2B). The statistical findings based on the I–V curve indicated that the suppressive effect of HHT on TMEM16A currents was mainly manifested in the outward currents, but did not affect the inward currents; the suppressive effect did not change the TMEM16A outward rectification characteristics (Figure 2C). Subsequently, we calculated the inhibitory efficiency of different HHT concentrations on TMEM16A currents and fitted the IC_50_ value of HHT to TMEM16A at 11.37 ± 1.68 μM using the Hill equation (Figure 2D). The statistical results showed that the maximum inhibition rate of HHT on TMEM16A currents reached 91.65 ± 5.90% (Figure 2E). Through the above whole-cell patch-clamp experiments, we confirmed that HHT is an effective TMEM16A inhibitor that suppresses TMEM16A currents in a concentration-dependent manner.

### 3.3. Key Binding Site of HHT and TMEM16A

**Figure 3:**
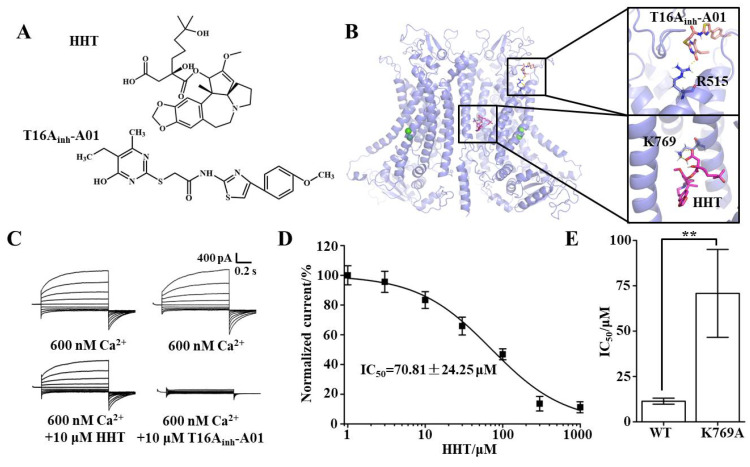
The binding site of HHT and TMEM16A. (**A**) Molecular structure of HHT and T16A_inh_-A01. (**B**) The putative binding site of HHT or T16A_inh_-A01 and TMEM16A. (**C**) Typical currents of HHT and T16A_inh_-A01 inhibited TMEM16A mutant (*n* = 5). (**D**) Dose-response curve for HHT inhibition of TMEM16A mutant currents (*n* = 5). (**E**) Statistical results of IC_50_ value of HHT to wild-type TMEM16A and mutant currents (*n* = 5, ** *p* < 0.01).

Molecular docking was performed to explore the putative binding sites of HHT and TMEM16A; the molecular structure of HHT is shown on Figure 3A. The results showed that the interaction between HHT and the K769 residue of TMEM16A is via hydrogen bonding (Figure 3B). Lysine was then mutated to alanine by site-directed mutagenesis. Whole-cell patch-clamp experiments were then performed with the mutant. Subsequently, the results showed that the whole-cell currents of the TMEM16A mutant were not inhibited by HHT, but the currents could be inhibited by T16A_inh_-A01 (the key binding site is R515, Figure 3B), which proved that the mutant is specifically sensitive to HHT (Figure 3C). The IC_50_ value of HHT for the TMEM16A mutant was 70.81 ± 24.25 μM, which was more than six times the IC_50_ value for wild-type TMEM16A (Figure 3D,E). Accordingly, we confirmed that K769 is the key binding site for HHT and TMEM16A.

### 3.4. TMEM16A Is a Potential Drug Target of HHT That Inhibits Lung Cancer Cell Proliferation

**Figure 4:**
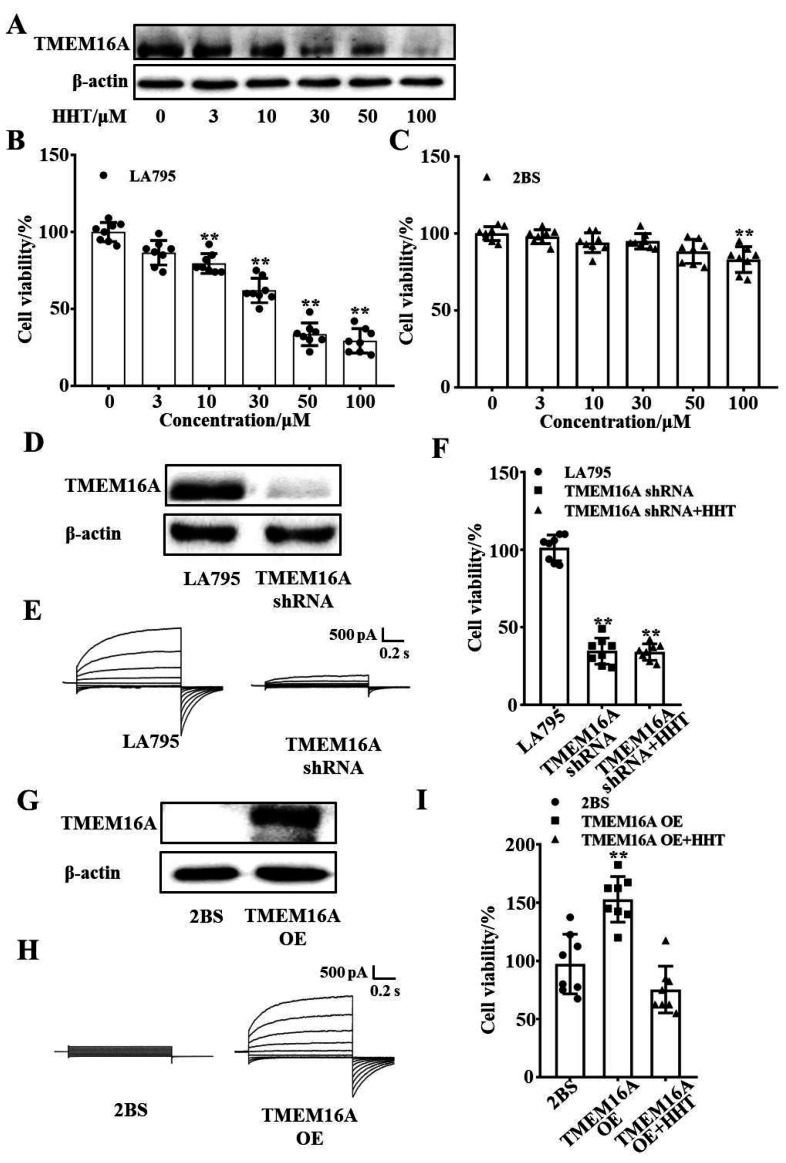
HHT inhibited lung cancer cell proliferation through inhibited TMEM16A. (**A**) TMEM16A protein expression treated with different concentrations of HHT for 24 h. (**B**,**C**) Inhibitory effect of HHT to the proliferation of LA795 and 2BS cells with different concentrations (*n* = 8). (**D**) Expression of TMEM16A protein in LA795 cells before and after shRNA transfection (*n* = 3, ** *p* < 0.01). (**E**) Typical whole-cell currents of TMEM16A in LA795 cells before and after shRNA transfection (*n* = 5). (**F**) Cell viability of LA795 cells before and after shRNA transfection (*n* = 8, ** *p* < 0.01). (**G**) Expression of TMEM16A protein in 2BS cells before and after TMEM16A transfection (*n* = 3). (**H**) Typical whole-cell currents of TMEM16A in 2BS cells before and after TMEM16A transfection (*n* = 5). (**I**) Cell viability of 2BS cells before and after TMEM16A transfection (*n* = 8, ** *p* < 0.01).

A western blot experiment was performed to detect the expression of TMEM16A protein in LA795 cells, which were incubated with different concentrations of HHT. The results showed that HHT incubation of LA795 cells for 24 h resulted in different degrees of reduction in the expression of TMEM16A (Figure 4A). CCK-8 experiments were performed with LA795 cells (endogenous, highly expressed TMEM16A) and 2BS cells (TMEM16A not expressed) to verify the inhibitory effects of HHT on the proliferation of lung cancer cells through the inhibition of TMEM16A expression. The results showed that HHT inhibited the proliferation of LA795 cells in a concentration-dependent manner but did not inhibit the proliferation of 2BS cells (Figure 4B,C). TMEM16A in LA795 cells was knocked out using shRNA (Figure 4D). TMEM16A currents almost disappeared (Figure 4E), and cell viability was significantly reduced after TMEM16A knockdown (Figure 4F). Correspondingly, TMEM16A expression increased after transfection of TMEM16A into 2BS cells (Figure 4G). TMEM16A currents were activated by 600 nM Ca^2+^ (Figure 4H). The cell viability increased after overexpression of TMEM16A, which was inhibited by HHT (Figure 4I). Thus, we confirmed that HHT suppresses the proliferation of LA795 cells by inhibiting TMEM16A expression.

### 3.5. TMEM16A Is a Potential Drug Target of HHT That Inhibits Lung Cancer Cell Migration

**Figure 5:**
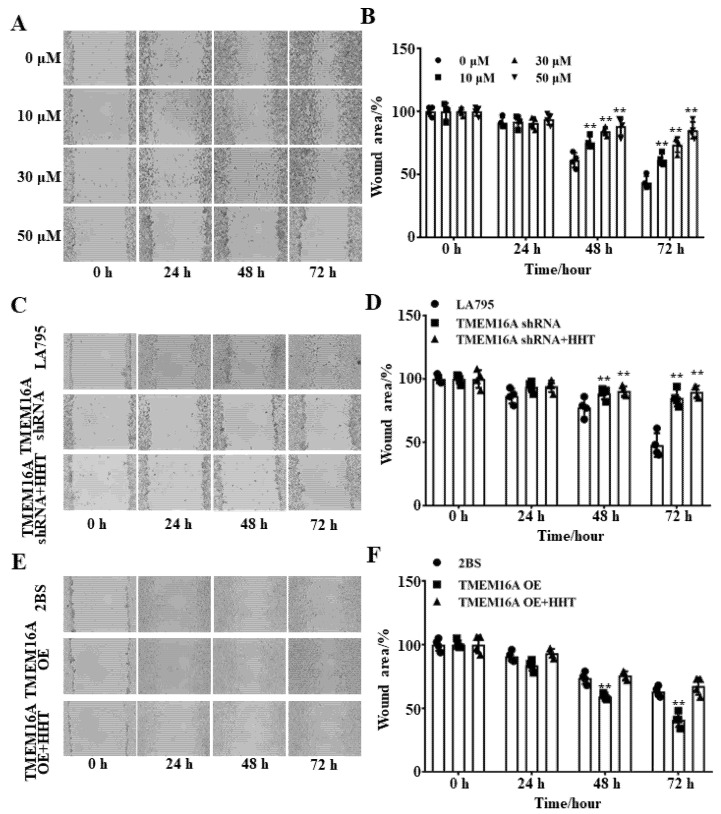
HHT inhibited lung cancer cell migration through inhibited TMEM16A. (**A**) Inhibitory effect of different concentrations of HHT on LA795 cell migration at 0, 24, 48 and 72 h (*n* = 3). (**B**) Statistical results of (**A**) (** *p* < 0.01). (**C**) The LA795 cell migration before and after shRNA transfection at 0, 24, 48 and 72 h (*n* = 3). (**D**) Statistical results of (**C**) (** *p* < 0.01). (**E**) The 2BS cell migration before and after TMEM16A transfection at 0, 24, 48 and 72 h (*n* = 3). (**F**) Statistical results of (**E**) (** *p* < 0.01).

Wound healing experiments were performed to test the inhibitory effects of HHT on LA795 cell migration. Figure 5A shows that the inhibitory effects of HHT against LA795 cell migration are more evident as the concentration of HHT increases. In addition, the inhibitory rates of the same concentration of HHT on LA795 cell migration increased with time. Therefore, the inhibitory effect of HHT on LA795 cells was observed to be concentration- and time-dependent (Figure 5B). Next, the migration of LA795 cells was significantly reduced after the endogenous TMEM16A was knocked down by shRNA (Figure 5C). In addition, HHT did not inhibit the migration of LA795 cells transfected with TMEM16A shRNA (Figure 5D). Correspondingly, overexpression of TMEM16A in 2BS cells obviously improved cell migration, which could be inhibited by HHT (Figure 5E,F). These findings prove that TMEM16A is a drug target of HHT, which inhibited LA795 cell migration.

### 3.6. TMEM16A Is a Drug Target of HHT That Promotes Lung Cancer Cell Apoptosis

**Figure 6:**
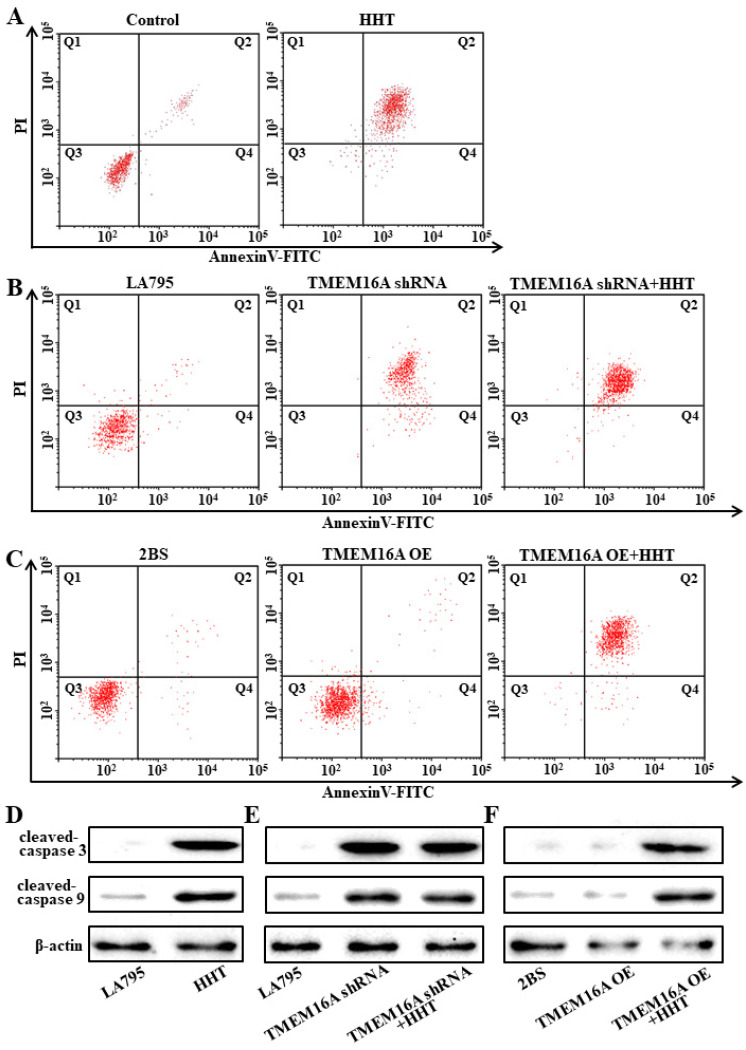
HHT promoted lung cancer cell apoptosis through TMEM16A. (**A**) Cell apoptosis results of LA795 cells incubated by 50 μM HHT for 24 h detected with Annexin-V assay (*n* = 3). (**B**) Cell apoptosis results of LA795 cells with TMEM16A shRNA transfection and added 50 μM HHT (*n* = 3). (**C**) Cell apoptosis results of 2BS cells with TMEM16A transfection and added 50 μM HHT (*n* = 3). (**D**–**F**) Expression of cleaved-caspase 3 and cleaved-caspase 9 with LA795 cells incubated by 50 μM HHT (**D**), or LA795 cells with TMEM16A shRNA transfection and added 50 μM HHT (**E**), or 2BS cells with TMEM16A transfection and added 50 μM HHT (**F**) (*n* = 3).

Annexin V and western blot assays were performed to detect apoptosis in HHT-treated cells. Results of the Annexin V analysis showed that the apoptosis rate of LA795 cells increased from 11.41 % to 79.63 % after incubating the cells with 50 μM HHT for 24 h (Figure 6A). At the same time, the levels of cleaved caspases 3 and 9 were increased (Figure 6D). Annexin V and western blot assays were also performed using LA795 cells after TMEM16A knockout. The results showed that the rate of apoptosis and the expression of apoptotic proteins in LA795 cells increased significantly after TMEM16A knockout. On this basis, it was considered that HHT did not promote apoptosis (Figure 6B,E). Correspondingly, overexpression of TMEM16A in 2BS cells did not promote apoptosis; however, incubation with HHT increased the rate of apoptosis and the expression of apoptotic proteins (Figure 6C,F). Therefore, TMEM16A is a drug target for HHT, which promotes cell apoptosis.

### 3.7. HHT Inhibits Lung Cancer Growth In Vivo

**Figure 7:**
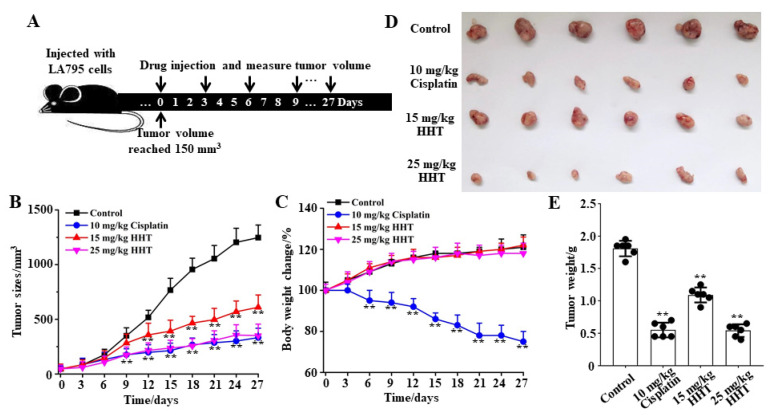
HHT inhibited the growth of lung adenocarcinomas in tumor xenograft mice. (**A**) Schematic diagram of the experimental protocol. (**B**) Tumor volume growth curve in different groups (*n* = 6). (**C**) Body weight change curve in different groups (*n* = 6). (**D**) Images of the tumor entity after 10 administrations of the drug (*n* = 6). (**E**) Statistical results of the stripped tumor weight in (**D**) (*n* = 6, ** *p* < 0.01).

A lung cancer xenograft mouse model was established by subcutaneously inoculating LA795 cells. Then, HHT was subcutaneously injected to test its inhibitory effects on tumor growth in vivo. Physiological saline and cisplatin (an anti-cancer chemotherapy drug) were used as the blank and positive controls, respectively. Statistical analysis and fitting of mice tumor volume growth curves showed that HHT significantly inhibited tumor volume growth in mice (Figure 7B). HHT (25 mg/kg) can achieve the same tumor inhibition efficiency as the maximum safe concentration of cisplatin [30]. At the same time, HHT did not reduce the body weight of mice like cisplatin (Figure 7C). After 10 administrations, the mice were sacrificed, and the tumors were dissected for weight measurement (Figure 7D). Results of the statistical analysis showed that the inhibition rates of 10 mg/kg cisplatin and 15 mg/kg and 25 mg/kg HHT against tumors were 69.6%, 40.5%, and 74.6%, respectively (Figure 7E). Therefore, we propose that HHT is a safe and efficient inhibitory drug for lung cancer.

### 3.8. Molecular Mechanism Underlying HHT-Mediated Inhibition of Tumor Growth In Vivo

**Figure 8:**
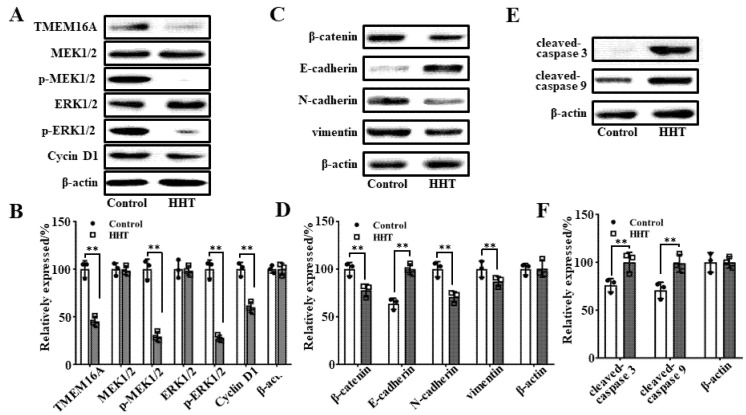
The molecular mechanism of HHT inhibited tumor growth. (**A**) Expression of TMEM16A, MEK1/2, phospho-MEK1/2, ERK1/2, phospho-ERK1/2, and cyclin D1 in 50 μΜ HHT incubated LA795 cells (*n* = 3). (**B**) Statistical results of (**A**) (*n* = 3, ** *p* < 0.01). (**C**) Expression of *β*-catenin, *E*-cadherin, *N*-cadherin, and vimentin in 50 μΜ HHT incubated LA795 cells (*n* = 3). (**D**) Statistical results of (**C**) (*n* = 3). (**D**) Expression of cleaved-caspase 3 and cleaved-caspase 9 in 50 μΜ HHT incubated LA795 cells (*n* = 3, ** *p* < 0.01). (**F**) Statistical results of (**E**) (*n* = 3, ** *p* < 0.01).

Western blot analyses were performed with tumor tissues to explore the molecular mechanism through which HHT inhibits the growth of lung cancer. The results showed that 50 μM HHT significantly reduced the expression of TMEM16A in LA795 cells (Figure 8A). HHT did not affect the expression of MEK1/2 and ERK1/2 in LA795; however, it reduced the phosphorylation of these proteins, which ultimately led to a decrease in cyclin D1 expression and arrested cells in the G0–G1 phase (Figure 8B). In addition, we detected key proteins related to cell invasion and apoptosis by western blotting. The results showed that the expression of β-catenin, *n*-cadherin, and vimentin was decreased and that of E-cadherin was increased in HHT-incubated LA795 cells (Figure 8C,D). Levels of the apoptotic proteins, cleaved caspases 3 and 9, were increased in HHT-incubated LA795 cells (Figure 8E,F). These findings indicate that HHT inhibited tumor cell growth by downregulating the protein expression of TMEM16A, which resulted in reduced cell proliferation and invasion and increased apoptosis.

## 4. Discussion

In this study, we confirmed that TMEM16A is a drug target for lung cancer and found that HHT is a lead therapeutic compound targeting TMEM16A in patients with lung cancer. Patch-clamp experiments showed that HHT inhibited TMEM16A expression in a concentration-dependent manner. The binding sites of HHT and TMEM16A were determined by molecular docking and site-directed mutagenesis experiments. Subsequently, the interaction between TMEM16A and cancer cell growth was studied using TMEM16A shRNA or overexpression. Finally, the inhibitory effect of HHT on lung cancer cells was explored in vivo and in vitro; the molecular mechanism underlying the inhibitory effects of HHT against lung cancer was explored by western blotting.

Several studies have shown that the TMEM16A gene is located in the 11q13 region of the human chromosome. TMEM16A expression is often amplified in cancers [18,31]. Thus, TMEM16A is highly expressed in some cancers [32,33]. In this study, we found that TMEM16A was highly expressed in lung cancer cells, whereas it was not expressed in normal lung cells. Furthermore, we confirmed that inhibiting the overexpression of TMEM16A in LA795 cells can suppress the proliferation and migration of cancer cells, whereas overexpressing TMEM16A in 2BS cells that typically have low TMEM16A expression promotes cell proliferation and migration. In addition, another inhibitor of TMEM16A, T16Ainh-A01, also showed an inhibitory effect on the growth of LA795 cells (Supplementary Materials Figure S2). We propose that TMEM16A is specifically overexpressed in lung cancer and plays a critical regulatory role in the proliferation and migration of cancer cells. In summary, TMEM16A is an ideal lung cancer biomarker and drug target.

Our data showed that HHT can both inhibit the ion channel activity of TMEM16A (Figure 2B) and down-regulate the expression of TMEM16A (Figure 4A). Molecular docking and site-directed mutagenesis experiments showed that HHT binds to K697 residues through the hydrogen bond to block the TMEM16A channel, which inhibited the ion channel activity of TMEM16A (Figure 3). One of the ways that HHT down-regulates the expression of the TMEM16A protein is through inhibiting the transcription process. The relative TMEM16A mRNA levels of LA795 cells decreased significantly after being incubated with 50 μM HHT for 24 h (Supplementary Materials Figure S1). In addition, HHT may reduce protein expression by inhibiting the translation extension, according to the literature [34].

Chemotherapy is one of the most commonly used methods for the treatment of lung cancer. However, many lung cancer patients develop drug resistance within a year of chemotherapy [35]; the most effective method to reverse drug resistance is multi-target combined treatment administration [36]. Therefore, it is particularly important to identify new lung cancer targets and targeted drugs. A combination of HHT and VCR has been proven to be effective; imatinib has a good therapeutic effect on leukemia [37,38]. As the target of HHT was different from that of other lung cancer drugs, it can be expected that the combination of HHT and other lung cancer drugs will produce enhanced therapeutic effects. This hypothesis needs to be verified in future studies.

In this study, we explored the molecular mechanism by which HHT inhibits lung cancer. The clinical effects of HHT on chronic myeloid leukemia are satisfactory. It also affects cell proliferation by preventing the synthesis of proteins and chromosomes [39,40]. However, few studies have investigated the effects of HHT against lung cancer, and its molecular mechanisms of action remain unclear. The results of this study confirmed that TMEM16A is an important receptor of HHT in lung cancer cell membranes. HHT blocks the cell cycle by inhibiting the phosphorylation of MEK1/2 and ERK1/2 in the MAPK signal transduction pathway by suppressing TMEM16A expression. At the same time, HHT suppresses cancer cell invasion and promotes apoptosis by inhibiting TMEM16A expression. These findings explain the anti-cancer mechanism of HHT and provide a research foundation for the development of HHT-related anti-cancer drugs.

HHT is a potential drug for lung cancer, with high safety and efficacy and low cost. HHT was approved for the treatment of adult chronic myeloid leukemia by the FDA in 2012 [41] as a subcutaneous injection twice a day for 28 days. HHT has been proven to have a good curative effect against leukemia as well as high biological safety over nearly 10 years of clinical application [42]. Our cell experiments showed that HHT had almost no side effects on the proliferation of 2BS cells (Figure 4B). Animal experiments also showed that HHT did not reduce the body weight of mice (Figure 7C), thereby verifying the biological safety of HHT. In addition, HHT is inexpensive and easy to produce. HHT is extracted from *Cephalotaxaceae* conifers, which are widely distributed in subtropical, evergreen, and broad-leaved forests [43]. The HHT extraction process is simple. High-purity HHT can be obtained through drying and grinding, chromatography, extraction, and recrystallization [44,45]. Therefore, the development and subsequent clinical application of HHT is convenient.

In view of the multiple advantages of HHT, the clinical implication of HHT used for the treatment of lung adenocarcinoma is important. First, TMEM16A is specifically expressed in lung cancer tissue [46]. HHT works through inhibiting TMEM16A and does not damage normal tissues that do not express TMEM16A, which means that the clinical side effects of HHT will be small. Second, the main targets of clinical lung cancer targeted drugs are EGFR, ALK, etc. [47,48]. The target of HHT is different from these; thus, HHT can solve the problem of clinical targeted drug resistance. Third, HHT has many years of clinical use experience as a treatment drug for CML and AML [24,48]. The pharmacokinetics and safety data of HHT are detailed. New uses for proven drugs can shorten the development cycle and maximize resource utilization.

In summary, TMEM16A overexpression is closely related to the growth of lung cancer cells. Thus, it may be an important drug target for the treatment of lung cancer. HHT suppressed the proliferation and migration of lung cancer cells by inhibiting TMEM16A channel activity. Therefore, targeting TMEM16A by the administration of HHT to inhibit lung cancer growth and development may represent an innovative strategy for treating the disease in the future.

## Supplementary Materials

The following are available online at https://www.mdpi.com/article/10.3390/ijms222010930/s1.
