# Supplementary material for: TMEM16A, a Homoharringtonine Receptor, as a Potential Endogenic Target for Lung Cancer Treatment
Source: Int J Mol Sci. 2021 Oct 10;22(20):10930. doi: 10.3390/ijms222010930 (PMC8535866; doi:10.3390/ijms222010930)
Supplement: Supplementary file 1 [file ijms-22-10930-s001.zip › ijms-1361306-supplementary.pdf]

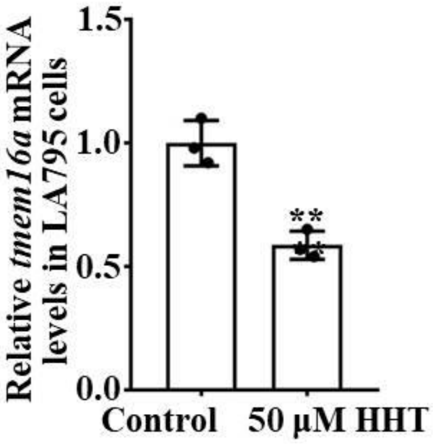

Supporting Data Figure S1. The relative *tmem16a* mRNA levels in LA795 cells treated with 50 μM HHT for 24 h (n = 3).

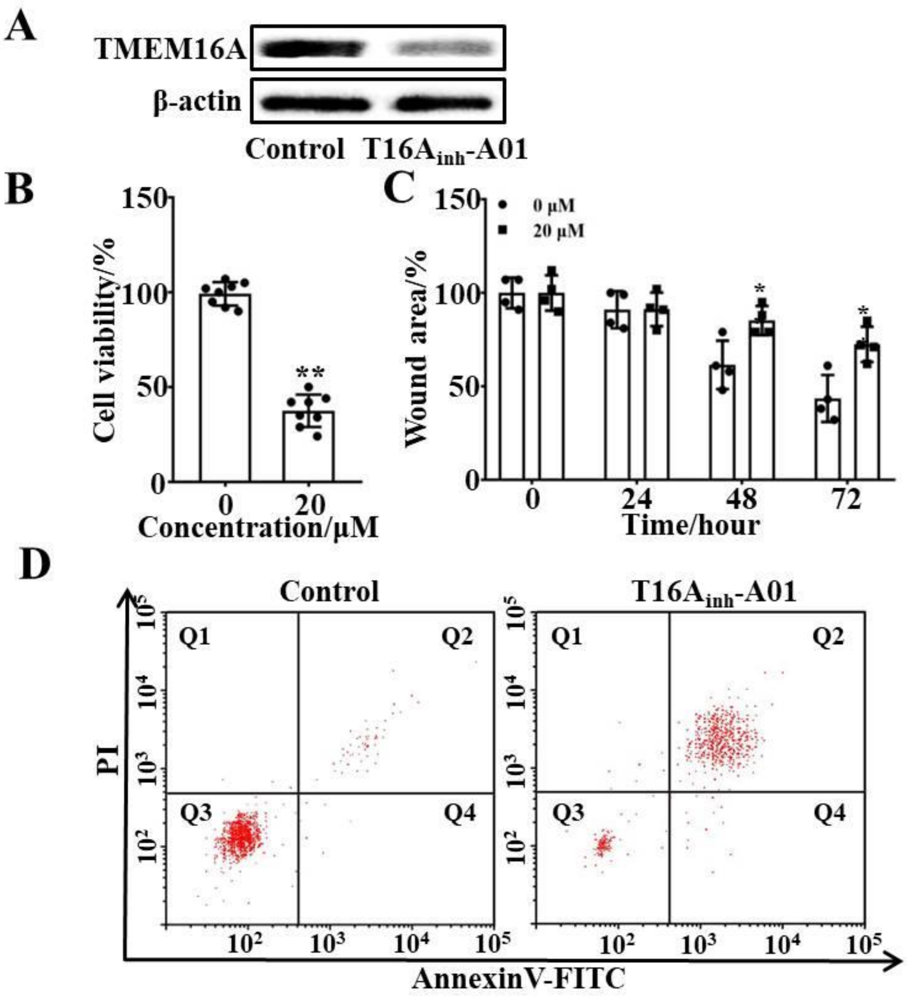

Supporting Data Figure S2. T16Ainh-A01 inhibited the proliferation and migration of LA795 cells and promoted apoptosis. (**A**) Expression of TMEM16A in 20 μM $T16A_{inh}$-A01 incubated LA795 cells (n = 3). (**B**) Inhibitory effect of $T16A_{inh}$-A01 to the proliferation of LA795 cells (n = 8). (**C**) Inhibitory effect of $T16A_{inh}$-A01 to the migration of LA795 cells (n=4). (**D**) Cell apoptosis results of LA795 cells incubated by 20 μM $T16A_{inh}$-A01 for 24 hours detected with Annexin-V assay (n = 3).
